# Efficacy of hypnosis/guided imagery in fibromyalgia syndrome - a systematic review and meta-analysis of controlled trials

**DOI:** 10.1186/1471-2474-12-133

**Published:** 2011-06-15

**Authors:** Kathrin Bernardy, Nicole Füber, Petra Klose, Winfried Häuser

**Affiliations:** 1 Department of Anaesthesiology, Intensive Care and Pain Therapy, Saarland University Hospital, Kirrberger Straße 100, D-66421 Homburg/Saar, Germany; 2 Department of Differential Psychology and Psychodiagnostics, Saarland University, Im Stadwald, D-66123 Saarbrücken, Germany; 3 Department of Internal Medicine V (Integrative Medicine), University of Duisburg-Essen, Kliniken Essen-Mitte, Am Deimelsberg 34a, D-45276 Essen, Germany; 4 Department of Internal Medicine I, Klinikum Saarbrücken, Winterberg 1, D-66119 Saarbrücken, Germany; 5 Department of Psychosomatic Medicine, Technische Universität München, Ismaninger Straße 22, D-81675 München, Germany

## Abstract

**Background:**

Recent systematic reviews on psychological therapies of fibromyalgia syndrome (FMS) did not consider hypnosis/guided imagery (H/GI). Therefore we performed a systematic review with meta-analysis of the efficacy of H/GI in FMS.

**Methods:**

We screened http://ClinicalTrials.gov, Cochrane Library, MEDLINE, PsycINFO and SCOPUS (through December 2010). (Quasi-) randomized controlled trials (CTs) comparing H/GI with controls were analyzed. Outcomes were pain, sleep, fatigue, depressed mood and health-related quality of life (HRQOL). Effects were summarized using standardized mean differences (SMD).

**Results:**

Six CTs with 239 subjects with a median of 9 (range 7-12) H/GI-sessions were analysed. The median number of patients in the H/GI groups was 20 (range 8-26). Three studies performed follow-ups. H/GI reduced pain compared to controls at final treatment (SMD -1.17 [95% CI -2.21, -0.13]; p = 0.03). H/GI did not reduce limitations of HRQOL at final treatment (SMD -0.90 [95% CI -2.55, 0.76]; p = 0.29) compared to controls. Effect sizes on fatigue, sleep and depressed mood at final treatment and follow-up and on pain and HRQOL at follow-up were not calculated because of limited data available. The significant effect on pain at final treatment was associated with low methodological and low treatment quality.

**Conclusion:**

Further studies with better treatment quality and adequate methodological quality assessing all key domains of FMS are necessary to clarify the efficacy of H/GI in FMS.

## Background

The key symptoms of fibromyalgia syndrome (FMS) are chronic widespread pain, fatigue (physical exhaustion and cognitive disturbances) and non-restorative sleep [1]. Besides these symptoms global multidimensional function ( = health related quality of life [HRQOL]) and tenderness are regarded to be the key domains of therapeutic trials in FMS [2].

Patients with FMS use a lot of pharmacological and non-pharmacological therapies resulting in high costs of health services [3]. Pharmacological and physical therapies are more frequently used than psychological treatments. Although hypnotic pain relief is among the oldest treatments for pain, interest in hypnotic treatments for chronic pain appears to rise only in the last decade [4]. In an internet survey only 3% of the respondents reported to use hypnosis to relieve FMS-symptoms [5].

A hypnotic procedure is used to encourage and evaluate responses to suggestions. When using hypnosis, one person (the subject) is guided by another (the hypnotist) to respond to suggestions [6]. The suggestions can be direct (traditional hypnosis) or permissive (Ericksonian hypnosis). Imagery is defined as a dynamic, psychophysiologic process in which a person imagines and experiences an internal reality in the absence of external stimuli. These images can be initiated by the patient or guided by a therapist (guided imagery) [7]. Both techniques aim to promote changes in subjective experience, alterations in perception, sensation, emotion, thought or behaviour by suggestion and/or imagination [6,7].

A recent qualitative review on hypnosis in chronic pain syndromes included only one study with hypnosis in FMS [8]. A recent systematic review on the efficacy of psychological therapies in FMS did not include studies with hypnosis/guided imagery [9]. Therefore we saw the need to perform a systematic review with meta-analysis of the efficacy of hypnosis/guided imagery compared to control therapies to reduce the key symptoms of FMS-patients of any age.

## Methods

The review was performed according to the PRISMA-statement (Preferred Reporting Items for Systematic Reviews and Meta-Analyses [10] and the recommendations of the Cochrane Collaboration [11].

### Protocol

Methods of analysis and inclusion criteria were specified in advance. We used the review protocol of our systematic review on cognitive behavioral therapies in FMS [12].

### Eligibility criteria

#### Types of interventions

Studies with hypnosis and guided imagery as an active treatment of primary interest for FMS were included. Hypnosis/guided imagery should use pain-related and/or pain- addressed suggestions and/or images. Studies with relaxation only (without trance induction or without the use of imagination) or with the combination of hypnosis with a defined pharmacological therapy as an active treatment of primary interest were excluded. Experimental studies (single session) with hypnosis/guided imagery were excluded.

#### Types of studies

A controlled design (controlled trials = CTs) was demanded. In case of multiple control groups we predefined the following order for comparison: Cognitive intervention (nonspecific elements of hypnosis/guided imagery such as education, emotional support, pure relaxation, suggestions without induction of hypnotic trance), treatment as usual, waiting list, active therapy (any defined pharmacological or non-pharmacological intervention other than hypnosis/guided imagery). The number of patients in each study arm should be > 5. The studies should be available as a full publication in a peer reviewed science journal.

#### Types of participants

Patients diagnosed with FMS based on defined criteria and of any age were included.

#### Types of outcomes measures

Studies should assess at least one key domain of FMS (pain, sleep, fatigue, HRQOL) [2]. Depressed mood was chosen for secondary outcome because depressive symptoms frequently occur in FMS-patients [1] and improving emotional status is one main target of hypnosis/guided imagery [6,7].

### Data sources and searches

The electronic bibliographic databases screened included http://ClinicalTrials.gov, Cochrane Central Register of Controlled Trials (CENTRAL), MEDLINE, PsycINFO and SCOPUS (through December 30, 2010). The search strategy for MEDLINE was as follows: (("Hypnosis"[Mesh] OR "Imagery (Psychotherapy)"[Mesh])) AND "Fibromyalgia"[Mesh] AND ((clinical[Title/Abstract] AND trial[Title/Abstract]) OR clinical trials[MeSH Terms] OR clinical trial[Publication Type] OR random*[Title/Abstract] OR random allocation[MeSH Terms] OR therapeutic use[MeSH Subheading])

The search strategy was adapted for the other databases. No language restrictions were made. In addition, reference sections of original studies were screened manually.

### Study selection

Two authors independently screened the titles and abstracts of potentially eligible studies identified by the search strategy detailed above (NF, PK). The full text articles were then examined independently by two authors to determine if they met the inclusion criteria (KB, WH).

### Data collection process

Two authors independently extracted the data using standard extraction forms (KB, NF). Discrepancies were rechecked and consensus achieved by discussion. If needed a third author reviewed the data to reach a consensus (WH).

We contacted all trial authors for further details of their methodology. The requests were answered by four authors. Where means or standard deviations (SDs) were missing, attempts were made to obtain these data through contacting four trial authors. Additional data were provided by three authors. Where SDs were not available from trial authors, they were calculated from t-values, confidence intervals or standard errors, where reported in articles. If these data were not available, the SD was substituted by the mean of the SDs of studies available which used the same outcome scale [12].

### Data items

The data of study setting, participants, inclusion and exclusion criteria, interventions, cotherapies, side effects reported and outcomes used for meta-analysis are listed in tables 1 and 2.

**Table 1:** Main characteristics of controlled studies with hypnosis/guided imagery in fibromyalgia syndrome

Author Country Year Setting Referral [Reference]	Mean age (years) Women %	Exclusion criteria	Diagnosis of FMS	Total study population Screened/randomized N (%) Total/Completing therapy N(%)	Treatment group Total/Completing therapy N(%)	Control group Total/Completing therapy N (%)	Outcomes used for metaanalysis
Alvarez 2007 Mexico University Hospital Rheumatology clinics [23]	44 yrs 100% women 100% Mexican mez-stizos	No other significant painful or chronic comorbidities	ACR	50/43 (86) 43/11 (26)	20/7 * (35)	23/4* (17)	Pain VAS 0-100 * Fatigue NA Sleep NA Depressed mood NA HRQOL: FIQ total * No follow- up

Castel 2009 Spain University Hospital Pain clinic [24]	44 yrs 95% women 100% white	Other severe chronic pain condition; severe psychopathology; moderate cognitive impairment;pending litigation	ACR 18-60 years At least 6 years education	NR/47 35/32 (91)	17/16 (94)	18/16 (89)	Pain NRS 0-10 Fatigue NA Sleep NA Depressed mood NA HRQOL FIQ total No follow-up

Grøndhal 2008 Norway General practice General practices [25]	23-54 yrs 75% women 100% white	Organic diseases Severe psychiatric disorder	Clinical diagnosis of chronic widespread pain	18/16 (89) 16/12 (75)	8/7 (88)	8/5 (63)	Pain NRS 1-7* (a) Fatigue NRS 1-7 * (a) Sleep NA Depressed mood NRS 1-7 * (a) HRQOL NA 1 year without control

Haanen 1991 Netherlands Rheumatology department regional hospital NR [26]	45 yrs 95% women NR	Organic diseases	Smythe Normal blood test	NP 40/37 (93)	20/17 (85)	20/20 (100)	Pain VAS 0-10 ** Sleep VAS 0-10 ** Fatigue VAS 0-10 *** Depressed mood NA HRQOL NA 12 weeks

Menziers 2006 USA University hospital Physicians and clinics [27]	50 yrs 98% women 90% white	Inflammatory rheumatic disease Major communicative disorder	Clinical diagnosis Age > 18 FIQ-Total > 20 MMSE score > 25	NP 48/NP	24/NR	24/NR	Pain VAS 0-10 Fatigue: NA Sleep NA Depressed mood NA HRQOL FIQ total 4 weeks

Rucco 1995 Italy Regional hospital [28]	38 yrs 100% women NR	Analgesic medication Major psychiatric disorder	ACR criteria Normal blood tests	NP 53/35 (66)	26/24 (92)	27/11 (41)	Pain VAS 0-10 Fatigue NA Sleep VAS 0-10 Depressed mood NA HRQOL NA No follow up

Abbreviations: * Data provided on request; (a) adjusted for baseline values; ** Median used for analysis; Standard deviation not reported; calculated from mean of other trials; *** Median not entered into meta-analysis; no calculation of standard deviation from other trials possible

FIQ = Fibromyalgia Impact questionnaire; HRQOL = Health-related quality of life; NA = Not assessed; NP = Not provided on request; NRS = Numeric Rating Scale; VAS = Visual Analogue Scale;

**Table 2:** Details on therapeutic techniques and co-therapies of the studies analysed

Author (Reference)	Types and duration of treatment in hypnosis/guided imagery group	Types and duration of treatment in controls	Comedication allowed Other cotherapies Side effects reported
Alvarez [23]	Ericksonian hypnosis, individual: Induction NR; 5 techniques could be used for utilisation: transformation of pain or emotion; metaphors; dissociation of pain; perceptions of other body sensations; pleasant imagery; posthypnotic suggestions of negative hallucinations and amnesia of pain 60 min, 6- 8 sessions over 6 months Total: 420 min	Cognitive intervention: Sham hypnosis, individual: General issues talking with the therapist; physical sign check list to verify that the patients were not in hypnotic trance * 60 min, 6- 8 sessions over 6 months Total: 420 min	NSAIDs, amitriptyline, physiotherapy kept stable during study * NR 4/20 with hypnosis drop out lack of efficacy 7/20 with sham hypnosis drop out lack of efficacy

Castel [24]	CBT plus standardised hypnosis; group: Induction by fixation and palpepral catalepsy; deepening by visualisation; suggestion of an analgesic stream eliminating pain and creating feelings of well-being; posthypnotic suggestions NR 90 min (of which 20 min hypnosis) for 12 weeks; daily home training with audiocasettes recommended Total: 1080 min	Active control: CBT; group. Information in FMS, cognitive restructuring, assertiveness training, behavioral goal setting, problems solving; 90 min (of which 20 min relaxation training) for 12 weeks Total: 1080 min Study arm treatment as usual not used for comparison	Analgesics, antidepressants, sedatives, myorelaxants NR NR

Grøndahl [25]	Standardised hypnosis, individual: Relaxation, visualisation of positive body experience; suggestion of increase of self-efficacy, posthypnotic suggestions NR 10 wks, 1/week, 30 min Total: 300 min	Treatment as usual: Medication and physiotherapy or chiropractic therapy Total Min NR	Medication and physiotherapy or chiropractic therapy NR NR

Haanen [26]	Standardised hypnosis, individual and daily audiocassette: Induction by arm levitation; suggestions of ego-strengthening, pain control and improvement sleep; posthypnotic suggestions NR 8 × 60 min over 3 months; daily home training Total: 360 min (without home training)	Active control Physical therapy: Massage and muscle relaxation 1-2 h/week over 12 weeks Total: 720-1440 min	Only paracetamol allowed NR NR

Menziers [27]	Standardised guided imagery, indvidual, at home: 3 standardised audio cassettes: Relaxation, signal breath to elicit relaxation; imagination of pleasant scene; suggestions of feeling of well-being and actions and behaviors free of FMS-symptoms 30 min daily for 6 weeks recommended Total: Median of 44 (range 37-136) exercises	Treatment as usual Total Min NR	Treatment as usual NR NR

Rucco [28]	Ericksonian hypnosis, individual: Metaphors for induction, Utilisation: identification and solution of intrapsychic conflicts; posthypnotic suggestions NR Frequency of sessions individualised over 6 months	Active control: Autogenic training in group Practice: 8 weeks, twice a week, 15 min; Recommendation of daily practice over 6 months Total: 240 min	No comedication allowed NR NR

* Details provided on request; NR = Not reported and not provided on request

### Risk of bias in individual studies and quality ratings

To ascertain the methodological quality of the eligible studies, two authors independently (KB, NF) rated eligible trials using a scale developed specifically for assessing the quality of psychological treatments for chronic pain [13]. Discrepancies were rechecked and consensus achieved by discussion. If needed a third author reviewed the data to reach a consensus (WH). The Quality Rating Scale is comprised of an overall quality score (0-35) consisting of two subscales. A treatment quality subscale (0-9) covers stated rationale for treatment, manualization, therapist training and patient engagement. Patient engagement was defined by checking for trance phenomena during hypnosis and/or execution of homework audiotape training. A design and methods quality subscale (0-26) covers inclusion/exclusion criteria, attrition, sample description, minimization of bias (randomisation method, allocation bias, blinding of assessment, equality for treatment expectations), selection of outcomes, length of follow-up, adequacy of statistical analyses (a priori power calculation, sufficient sample size, adequate data analysis and summary statistics, intention to treat analysis) and choice of control. We assumed a sample size of at least 10 per treatment arm to be sufficient. We defined scores 0-2 to indicate a poor, scores 3-5 an average and scores 6-9 an excellent treatment quality and scores 0-12 indicating a low, scores 13-19 a medium and scores > 19 a high methodological quality. Interrater reliability was calculated for both subscales by intra-class correlation coefficients (ICC).

### Summary measures

Meta-analyses were conducted using RevMan Analyses software (RevMan 5.0.24) of the Cochrane collaboration [14]. Standardized mean differences (SMD) were calculated by means and SDs for each intervention. Examination of the combined results was performed by a random effects model (inverse variance method), because this model is more conservative than the fixed-effects model and incorporates both within-study and between-study variance [15]. SMD used in Cochrane reviews is the effect size known as Hedges (adjusted) g. We used Cohen's categories to evaluate the magnitude of the effect size, calculated by SMD, with g > 0.2-0.5 = small effect size, g > 0.5-0.8 = medium effect size, g > 0.8 = large effect size [16].

### Planned methods of analysis

Heterogeneity was tested using the I² statistics with I² values above 50% indicate substantial heterogeneity. Tau² was used to determine how much heterogeneity was explained by subgroup differences [11].

### Risk of bias across studies

Potential publication bias (i.e. the association of publication probability with the statistical significance of study results) was investigated the Egger test, in which the standardized effect size (effect size calculated by standard error) is regressed on precision (inverse of standard error). The intercept value is an estimate of asymmetry of funnel plot. Positive values (> 0) indicate higher levels of effect size in studies with smaller sample sizes [17].

### Additional analyses

#### Subgroup and sensitivity analysis

If there were at least three studies available, subgroup analyses were prespecified for type of psychological therapy (hypnosis and guided imagery; hypnosis/guided imagery with and without home training with audiotapes) and type of control group. These subgroup analyses were also used to examine potential sources of clinical heterogeneity.

We decided post-hoc to perform a sensitivity analysis of studies without calculated values (median instead of mean; SD calculated from other studies; adjusting for baseline values).

#### Metaregression analyses

We a priori decided to metaregress SMDs with the treatment and methodological quality score for potential sources of heterogeneity. We post - hoc decided to metaregress SMDs with the number of the participants of the studies. Meta-regression was performed using the mixed effects model. Tau² variance was calculated by the method of unrestricted maximum likelihood by Comprehensive Metaanalysis software [18].

## Results

### Results of search

**Figure 1:**
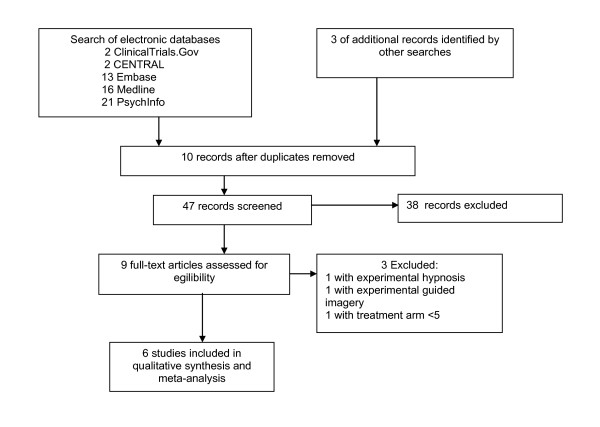
**PRISMA flow diagram**.

The search of literature yielded 57 hits. After excluding studies based on information presented in study abstracts, nine complete study reports were considered in more detail. One double published study with experimental guided imagery [19,20], one study with experimental hypnosis [21] and one study with two patients in each treatment arm [22] were excluded. Finally six studies were included in qualitative analysis [23-28] (see figure 1).

### Study characteristics

#### Setting, referral and exclusion criteria

Four studies were conducted in Europe and one study each in USA and Mexico. Patients were recruited by registers of hospitals, referral (general practitioner, rheumatologist, departments of hospitals) and local self-help groups. Five studies were conducted in hospitals (university, district hospital) and one study in a general practitioner office. All studies were single center based. One study did not report the exclusion criteria. All other studies included only adult patients (> 18 years) and excluded patients with major somatic and/or mental diseases. One study excluded patients with litigation/pension associated with FMS and one study excluded patients who were taken analgetisic medication (see table 1).

FMS was diagnosed in three studies by the criteria of the American College of Rheumatology [29], in one study by the Smythe criteria [30] and in two studies by clinical (not further specified) criteria. No study reported the frequency of mental disorders.

#### Participants

Two studies reported the percentage of persons screened who were subsequently randomised with a median of 87 (range 86-89) %. The median of the mean age of the participants was 44 (range 38- 50) years. The median of the percentage of women was 96 (range 75-100) %. Six studies reported the race of the patients. The median of the percentage of Caucasians was 95 (0-100) %. One Mexican study included 100% mestizos.

The median of the patients under hypnosis/guided imagery in the studies was 20 (range 8-26) and of controls was 22 (range 8-27) (see table 1). The total number of patients with hypnosis/guided imagery was 115 of which a median percentage of 88 (range 35-94) % completed therapy. The total number of patients in the control groups was 124 of which a median percentage of 63 (range 17-100) completed therapy (z = -0.5, p = 0.7).

#### Interventions

Five studies offered hypnosis: Three studies with direct hypnosis of which one was combined with cognitive-behavioral therapy, two studies with indirect [Ericksonian] hypnosis). One study offered guided imagery. Four studies with hypnosis explicetly mentioned the use of mental images. All but one study used suggestions and/or images which were directly addressed to the pain experience. All studies used pain-related suggestions. The study with guided imagery used suggestions. Hypnosis/guided imagery were delivered in five studies as individual therapy and in one study as group therapy. Hypnosis/guided imagery were offered in five studies by face-to face (life), in one study by audiotapes. Three studies recommended daily training at home with audiotapes. The median number of sessions with a therapist was nine (range 7-12). The median of hypnosis/guided imagery delivered by a therapist was 390 (range 300-1080 min). The number of sessions in one trial with Ericksonian hypnosis was individualized. Median and range of the number of sessions of this study were not reported.

In one study controls received a cognitive intervention (sham hypnosis), in three studies active therapy (cognitive behavioral therapy, physical therapy, autogenic training) and in two studies treatment as usual (see table 2).

Three studies performed a follow-up of which one (after one year) was without controls because the controls had switched to hypnotherapy. The median of follow-up of the other two studies was 8 (range 4-12) weeks.

#### Outcomes

Pain was assessed in all studies, sleep in two and fatigue and depressed mood in one study each by visual or numeric scales. HRQOL was assessed in three studies by the Fibromyalgia Impact Questionnaire. Two authors provided additional outcomes on request. One author did not provide the means and standard deviations of his study in which medians and ranges had been presented (see table 1).

No study assessed predefined response rates (e.g. percentage of patients with 30% pain reduction). Only one study reported the number of patients who dropped out because of lack of efficacy. No study reported on side effects.

### Quality ratings of trials

Three authors provided additional information on methods on request (see tables 3 and 4). The ICC using absolute agreement for the two raters was 0.96 (F = 3.4) for the treatment quality and 0.97 (F = 3.4) for the design quality subscale.

**Table 3:** Treatment quality of studies with guided imagery/hypnosis in FMS

Author (Reference)	Treatment content	Treat-ment duration	Manualisa-tion of treatment	Adherence to manual	Therapist training	Client engagement	Sum
Alvarez [23]	Adequate (2)	Reported (1)	Partial (1) *	Partial (1) *	Adequate (2) *	Adequate (1)*	8

Castel [24]	Adequate (2)	Reported (1)	Partial (1)	Inadequate (0)	Inadequate (0)	Inadequate (0)	4

Grøndahl [25]	Adequate (2) *	Reported (1)	Adequate (2) *	Partial (1) *	Adequate (2) *	Adequate (1)	9

Haanen [26]	Adequate (2)	Reported (1)	Partial (1)	Inadequate (0)	Inadequate (0)	Inadequate (0)	4

Menziers [27]	Adequate (2)	Reported (1)	Adequate (2)	Adequate (2)	Inadequate (0)	Adequate (1)	8

Rucco [28]	Inadequate (0)	Not reported (0)	Inadequate (0)	Inadequate (0)	Inadequate (0)	Inadequate (0)	0

* Data provided on request

**Table 4:** Methodological quality of studies with guided imagery/hypnosis in FMS

Author (Reference)	**Alvarez **[23]	**Castel **[24]	**Grøndhal **[25]	**Haa-nen **[26]	**Men-ziers **[27]	**Rucco **[28]
Sample criteria	Adequate (1)	Adequate (1)	Adequate (1)	Adequate (1)	Adequate (1)	Inadequate (0)

Evidence that sample criteria were met	Adequate (1)	Adequate (1)	Inadequate (0)	Adequate (1)	Adequate (1)	Inadequate (0)

Report of attrition	Adequate (2)	Partial (1)	Adequate (2)	Adequate (2)	Adequate (2)	Partial (1)

Rates of attrition	Adequate (1)	Adequate (1)	Adequate (1)	Adequate (1)	Adequate (1)	Adequate (1)

Sample characteristics	Adequate (1)	Adequate (1)	Inadequate (0)	Adequate (1)	Adequate (1)	Adequate (1)

Group equivalence	Adequate (1)	Inadequate (0)	Inadequate (0)	Adequate (1)	Adequate (1)	Adequate (1)

Adequacy of randomisation	Adequate (2)*	Inadequate (0)	Adequate (2)*	Inadequate (0)	Inadequate (0)	Inadequate (0)

Concelament of treatment allocation	Adequate (1)*	Inadequate (0)	Adequate (1)*	Inadequate (0)	Inadequate (0)	Inadequate (0)

Blinding of assessor	Adequate (1)*	Inadequate (0)	Adequate (1)*	Inadequate (0)	Inadequate (0)	Inadequate (0)

Treat-ment expectations	Inadequate (0)	Inadequate (0)	Inadequate (0)	Inadequate (0)	Inadequate (0)	Inadequate (0)

Justifications of outcomes	Adequate (1)	Adequate (1)	Adequate (1)	Adequate (1)	Adequate (1)	Adequate (1)

Validity of outcomes for context	Adequate (2)	Adequate (2)	Adequate (2)	Adequate (2)	Adequate (2)	Adequate (2)

Reliability and sensitivity to change of outcomes	Adequate (2)	Adequate (2)	Adequate (2)	Adequate (2)	Adequate (2)	Adequate (2)

Follow up of at least 6 months	Inadequate (0)	Inadequate (0)	Inadequate (0)	Inadequate (0)	Inadequate (0)	Inadequate (0)

Power calculation	Inadequate (0)	Inadequate (0)	Inadequate (0)	Inadequate (0)	Inadequate (0)	Inadequate (0)

Sufficient sample size	Inadequate (0)	Adequate (1)	Inadequate (0)	Adequate (1)	Adequate (1)	Adequate (1)

Adequate data analysis	Adequate (1)	Adequate (1)	Adequate (1)	Adequate (1)	Adequate (1)	Adequate (1)

Adequate reporting of summary statistics	Adequate (1)	Adequate (1)	Adequate (1)*	Adequate (1)	Adequate (1)	Inadequate (0)

Intention to treat analysis	Inadequate (0)	Inadequate (0)	Inadequate (0)	Adequate (1)	Inadequate (0)	Inadequate (0)

Control group	Adequate (2)	Adequate (2)	Adequate (2)	Adequate (2)	Adequate (2)	Inadequate (0)

Sum	20	14	17	17	16	10

* Data provided on request

The median of the treatment quality score was 6 (range 1-9). One study had a poor, two studies had a medium and three studies had an excellent treatment quality score (see table 3).

The median of the methodological quality score was 16 (range 10-20). One study had a poor, four studies had a medium and one study had an excellent methodological quality score (see table 4).

### Synthesis of results

#### Overall meta-analysis

**Figure 2:**
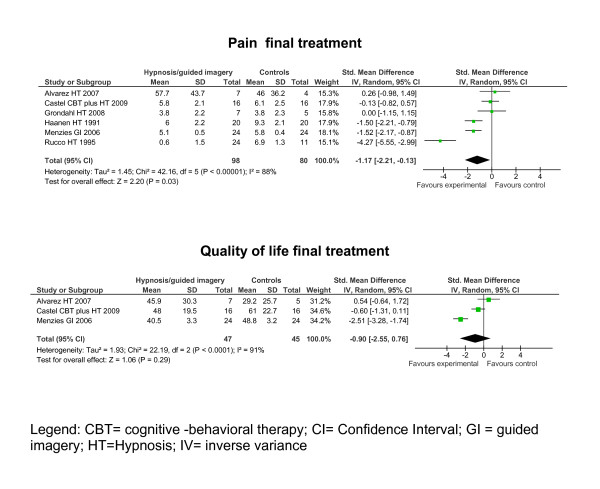
**Forest plots of the effect estimates (standardised mean differences) of hypnosis/guided imagery versus controls on outcomes at final treatment**.

The means, SDs, sample sizes and effect estimates at posttreatment and at follow-up of the remaining eight studies can be seen in the forest plots (see figure 2).

The data are reported as follows: (SMD [95% Confidence interval]). Hypnosis/guided imagery reduced pain (-1.17 [-2.21, -0.13]) compared to controls at final treatment. Hypnosis/guided imagery did not reduce the limitations of HRQOL -0.90 [-2.55, 0.76] compared to controls at final treatment. Based on Cohens' categories the effect on pain at final treatment was large.

Because of limited data available effect sizes on sleep, fatigue and depressed mood were not calculated at final treatment and not for all outcomes at follow-up (see table 1). Two studies reported that hypnosis was superior to controls in reducing sleep disturbances at final treatment [26,28]. Two studies reported that hypnosis/guided imagery was superior to controls in reducing pain at follow-up [26,27]. One study reported that hypnosis was superior to controls in reducing fatigue at final treatment and at follow-up [26]. In one study hypnosis was not superior to therapy as usual at posttreatment in reducing depressed mood [25].

#### Potential publication bias

In the Egger's test the intercept of the effect size on pain was 0.13 (95% CI -8.7, 9.0) with t = 0.03 (two-tailed p = 0.97) and thus not indicative for a publication bias.

#### Risk of bias across studies and subgroup analysis

There was substantial heterogeneity in the outcome pain at final treatment (see table 5). Subgroup analyses were not calculated, because < 4 studies for subgroups were available.

**Table 5:** Effect sizes of hypnosis/guided imagery on selected outcome variables

Outcome title	Number of studies	Number of patients	Effect size (SMD [95% CI])	Test for overall effect p-value	Heterogeneity I² [%]; Tau²
**Final treat-ment**					

01 Pain	6	178	-1.17 [-2.21, -0.13]	0.03	88; 1.45

02 Fatigue	1	12	Not calculated		

03 Sleep	2	66	Not calculated		

04 Depressed mood	1	12	Not calculated		

05 HRQOL	3	92	-0.90 [-2.55, 0.76]	0.29	91; 1.93

**Follow up**					

01 Pain	2	88	Not calculated *		

02 Fatigue	1	40	Not calculated		

03 Sleep	1	40	Not calculated		

04 Depressed mood	0	0			

05 HRQOL	1	48	Not calculated		

**Abbreviations: **HRQOL: Health-related quality of life; SMD: Standardised mean difference; CI: Confidence interval

* Standard deviation of one study not available

#### Sensitivity analysis

The effect on pain at final treatment was no more significant after removing the two studies in which some values were calculated by the authors of this review(-1.38 [-2.95, 0.20]; I² = 92%; p = 0.09).

#### Metaregression analyses

Simple linear regressions showed that treatment quality (ß = -3.6, p < 0.0001), and methodological quality (ß = -8.7, p < 0.0001)) were significantly negatively associated with the effect size on pain at final treatment. Sample size was not associated with the effects size on pain at final treatment (ß = 0.42, p = 0.65).

## Discussion

### Summary of evidence

The evidence of the efficacy of hypnosis/guided imagery to reduce pain at final treatment was not robust against risks of methodological bias and was associated with low methodological study quality.

### Applicability of evidence

The study settings of all levels of care and the study samples with a preponderance of middle aged women are representative for clinical FMS populations in America and Europe.

#### Agreements with other systematic reviews

The German FMS guideline group concluded by a qualitative systematic review that hypnosis/guided imagery was superior to controls in reducing pain, sleep disturbances and fatigue [31]. Our update of the literature with inclusion of recently published studies and quantitative analysis of outcomes supports that hypnosis/guided imagery was effective in relieving pain and sleep problems. A meta-analytic confirmation of the efficacy on fatigue was not possible because of the methodological problems outlined above. In contrast to a recent qualitative review on psychological therapies in FMS which described "mild effects" of hypnosis/guided imagery on FMS-symptoms [32] we found large effects on pain and medium effects sizes on sleep at final treatment and at follow-up.

## Limitations

We decided to pool studies with hypnosis and guided imagery because of their similaries regarding theoretical assumptions and therapeutic technics. But not all hypnosis involves guided imagery, and guided imagery does not necessarily result in a hypnotic state. The intended subgroup analysis of hynosis and guided imagery was not possible due to limited data.

In contrast to a recent Cochrane review on psychological therapies in chronic pain [33] we included studies with less than 10 participants in each study arm because of the limited number of studies with hypnosis/guided imagery available. Metaregression demonstrated no significant correlation between the magnitude of effect size on pain at final treatment and sample size. Thereforefore the positive effect on pain is robust against small sample size in our analysis.

Although every effort was made to obtain missing data on study design from authors, it was not possible in every case to obtain them. Therefore the study quality might be underestimated in some trials.

Some major risks of bias listed in Cochrane reviews (adequacy of randomisation, concealment of treatment allocation, blinding of the outcome assessor, ITT-analysis) [10] were present in the majority of studies. Metaregression analysis demonstrated that the positive effect on pain was associated with low methodological study quality.

We substituted one missing SD despite the small sample sizes and substantial heterogeneity. Furthermore we adjusted the outcomes posttreatment by baseline values because of baseline differences of one study. We demonstrated by sensitivity analyses that by removing these two studies only a statistical trend (p < 0.1) of the effect of hypnosis/guided imagery on pain at final treatment was detectable.

There was significant between-study heterogeneity for the outcome pain. To address this limitation we used a random-effects model.

It should be emphasized that all analyses might be underpowered due to the limited number of studies.

Adverse events were not reported. Therefore no definitive statement on the safety of hypnosis/guided imagery in FMS is possible.

Responses in studies with chronic pain patients are frequently not Gaussian, but with a split between responders and non-responders. No study assessed predefined response rates (e.g. 30% pain reduction). Therefore IMMPACT response outcomes [34] could not be calculated.

## Conclusions

### Implications for clinical practice

Because of the methodological limitations of the studies with hypnosis/guided imagery outlined above we cannot fully recommend hypnosis/guided imagery for FMS therapy. The use of hypnosis/guided imagery as an adjunct to efficacious pharmacological and non-pharmacological treatments had been recommended by the German interdisciplinary guideline on FMS based on expert consensus [31]. Regular home training by audiotapes with hypnotic suggestions and guided imaginations could be useful.

### Implications for research

Further studies with multi-site recruitment producing adequate sample sizes are necessary to allow for stronger tests of treatment efficacy and for examination of individual (e.g. gender, age, hypnotisability) differences in treatment response. Moreover the appropriate methods (live or audiotape therapy or combination of both types) and the optimal dosage need to be determined. Predictors of positive treatment outcomes, e.g. suggestibility and treatment expectations, should be explored [32].

The methodological quality of further studies could be improved by the following issues: The key domains of FMS should be assessed by a core set of outcome measures. Response rates should be measured [34]. Recommendations on the quality of the treatment delivered and study design should be followed [13].

## Competing interests

Dr. Häuser received honoraria for educational lectures by Eli-Lilly, Janssen-Cilag, Mundipharma and Pfizer and a congress travel grant by Eli-Lilly. Dr. Bernardy received a congress travel grant by Pfizer. The other authors have no competing interests to declare.

## Authors' contributions

All authors searched the literature, exctracted and analysed the data. All authors had been involved in drafting the manuscript or revising it critically for important intellectual content and gave final approval of the version to be published.

## Authors' information

WH was responsible for the coordination of the German interdisciplinary guideline on the management of fibromyalgia syndrome. He is a trainer in hypnosis. KB and PK were the scientific secretaries of the German guideline group. KB is a licensed psychologist for cognitive-behavioral therapy.

## Pre-publication history

The pre-publication history for this paper can be accessed here:

http://www.biomedcentral.com/1471-2474/12/133/prepub
